# Key Determinants of Anemia among Youngsters under Five Years in Senegal, Malawi, and Angola

**DOI:** 10.3390/ijerph17228538

**Published:** 2020-11-18

**Authors:** Chris Khulu, Shaun Ramroop

**Affiliations:** School of Mathematics, Statistics and Computer Science, University of KwaZulu-Natal, Private Bag X01, Scottsville 3209, South Africa; ramroops@ukzn.ac.za

**Keywords:** anemia, hemoglobin, chi-square test, gamma measure, GLMM

## Abstract

Anemia is characterized as a condition where there is a deficient number of hematocrit, hemoglobin, or red cells in the human body. This condition affects most youngsters under five years old and pregnant women. The fundamental goal of this paper is to investigate anemia, recognize its determinants, and propose critical proposals to achieve 2030 Sustainable Development Goal with a focus on Senegal, Malawi, and Angola. This research utilized 2016 nationally representative information from Senegal, Malawi, and Angola, which involved collecting data on the demographic and health of the populaces. The Demographic and Health Survey information from Senegal, Malawi, and Angola was then merged to create a pooled sample. This statistical technique enables to generalize and compare the results. A generalized linear mixed model was utilized to decide the factors correlated with anemia among youngsters under five years in Senegal, Malawi, and Angola. The analysis was performed in SPSS and SAS software. A generalized linear mixed model results showed that, compared to youngsters aged less than 12 months, youngsters in the age interval 13–23, 24–35, 36–47, and 48–59 months are more likely to be affected by anemia (OR = 1.419, 2.282, 3.174 and 4.874 respectively). In this study, seven factors were included in the final model. However, only five were found to be significant in explaining anemia at the 5% level of significance. The generalized linear mixed model identified youngster’s age, gender, mother’s level of schooling, wealth status, and nutritional status as determinants of anemia among youngsters under five years in Senegal, Malawi, and Angola.

## 1. Introduction

Anemia is characterized as a condition where there is a deficient number of hematocrit, hemoglobin, or red cells in the human body [1,2]. This condition affects most youngsters under-five-year old and pregnant women [3,4].

Approximately half of the youngsters’ populations between the ages of 0–5 years are anemic and one-third of pregnant women are anemic [5]. Mild anemia, moderate anemia and severe anemia are the three types of anemia. Mild anemia is associated with the hemoglobin concentration level in the interval of 10.0–10.9 g/dL.

Moderate anemia is associated with the hemoglobin concentration level in the interval of 7.0–9.9 g/dL, whereas severe anemia relates to the level of hemoglobin concentration that is less than 7.0 g/dL. This individual classification applies to both youngsters under five years and pregnant women.

3.3% of youngsters age 6–59 months in Africa are suffering from severe anemia, this estimate is two times the global estimate of youngsters suffering from anemia [6].

Current literature reveals that improving the parent level of education and household income reduces a child risk of suffering from anemia [1,2,4,7]. Age of the child, household size, and being exposed to malaria conditions increase the chances of suffering from anemia [8,9,10].

A study conducted by Ngwira & Kazembe [11] suggested that Malawian policymakers and government need to address poverty, maternal anemia and malnutrition in an attempt to reduce youngsters under five years mortality. Also, the research by Brabin et al. [12] revealed that improving malaria and anemia control in pregnancy would reduce the child’s risk of suffering from anemia conditions. In contrast, a study by Ntenda et al. [13] showed that mother’s level of education, household wealth status, youngster experiencing fever, and stunting are positively associated with anemia for Malawian youngsters under than five years.

Anemia and malaria are the most contributing diseases to under five/pre-school aged youngsters in Angola. Approximately 60% and 19% of pre-school aged youngsters are suffering from anemia and malaria respectively in Angola [14]. A study conducted by Sousa-Figueiredo et al. [15] defined anemia as a severe public health threat among youngsters under five years in Angola. The findings of the study identified the age group and sex of the youngster as a key determinant of anemia.

Results from the study conducted by Diouf et al. [16] in Senegal showed that improving the mother’s level of literacy and the consumption of animal protein (meat, fish, and eggs) reduce the youngster’s hazard of suffering from anemia. In contrast, the study conducted by Tine et al. [17] in Senegal showed that malaria parasitaemia, sickle cell disorders, alpha-thalassemia, stunting, age interval of 2 to 4 years and age greater than five years are significantly associated with anemia among youngsters under 10 years. In addition, a study reviewing observational studies of 15 countries, including Malawi and Senegal, by McCuskee et al. [18] concluded that covariates, such as age, type of residence, socio-economic status, and maternal education, tend to be associated with anemia.

There is a severe public health issue among youngsters under five years of age in Senegal, Malawi, and Angola. This is one of the conclusions drawn from the report released by the World Health Organization (WHO) [6]. In addition, youngsters are observed as future leaders, hence an increase in youngsters’ mortality rate is causing threats to the economy and long-term sustainability of the country.

The African continent is among the continents that have the high mortality rate among youngsters under five years. Comparing the current mortality rate of youngsters under five years old in Senegal, Malawi, and Angola to the 2030 Sustainable Development Goal (SDG) mortality rate, these countries are expected to decrease their mortality rate of under-five-year-old youngsters by at least half [19].

This is a very stretched and demanding target considering that the African region is a developing region with inadequate resources. A study conducted by Fancony et al. [20] to understand the factors associated with anemia and the interventions implemented to prevent this condition showed that the progress of implementing interventions is slow and this may be due to different reasons including the economy. This indicates the necessity of collaboration to ensure the implementation of interventions is achieved. Therefore, the fundamental goal of this paper is to investigate anemia, recognize its determinants, and propose critical interventions to achieve 2030 Sustainable Development Goal targets in Senegal, Malawi, and Angola, while considering limited resources.

The significance of this study underpins proposing insightful strategies that will enable Senegal, Malawi, and Angola to achieve the SDG target.

## 2. Methods and Materials

### 2.1. Source of Data

The data employed are from 2016 nationally representative information from Senegal, Malawi, and Angola. The survey collected data on the demographic and health of the populace. The data were requested from the DHS micro program. There was no ethical approval required since this study is secondary data analysis.

Demographic and Health Survey of Angola was collected beginning of October 2015 until the end of March 2016 to obtain demographic, socio-economic, health and other information. A household sample of n = 16,109 was selected and it was made up of 14,379 women aged between 15 and 49 and 5684 men aged between 15 and 54. The success rate achieved for interviewing men and women was 94% and 96% respectively.

The 2015–2016 Malawi Demographic and Health Survey had a sample size of 27,516 households of which 26,564 was successfully interviewed. The method used to determine the sample population was stratification completed in two stages. The primary stage of sampling was made up of 173 standard enumeration areas in the urban settings and 677 in standard enumeration areas rural settings. At the secondary stage, 30 families for every urban cluster and 33 for every rural cluster were chosen with an equal probability systematic selection.

The 2016 Senegal Demographic and Health Survey was executed and conducted by the National Agency of Statistics and Demographics to respond to public health challenges, community challenges, well-being challenging and to monitor the progress of implemented measures. A sample of 3527 men aged between 15 to 59 and 8865 women aged between 15 and 49 was interviewed to collect the information on demographics and health. A sum of 5722 youngsters under five years was weighted to decide their nutritional status and 5239 youngsters aged between 6 and 59 months were tried for anemia.

The Demographic and Health Survey data from Senegal, Malawi, and Angola was merged to create a pool sample. The created pool sample was then used for all the analyses conducted on this study. The advantage of using pooled sample when comparing different data set includes the ability to generalize the results. A similar method was used by Takele et al., Subramanian et al., Neuman et al., and Khulu and Ramroop [21,22,23,24]. The summary statistic of sample size by country is shown in Table 1 and Table 2.

### 2.2. Variables

#### 2.2.1. Outcome Variable

The response variable (youngsters under five year’s anemia status) was obtained from the anemia level variable in the DHS dataset for all three countries. The results of the hemoglobin level from the blood test was used to decide the level of anemia among youngsters under five years. Blood specimens were collected from all eligible children under five years whose parents voluntarily consented to be tested.

After obtaining blood samples, hemoglobin was carried out using a portable Hemocue analyser. In this study, youngsters under five years hemoglobin level was employed to classify mild, moderate, and severe anemia levels.

Youngsters under five years with hemoglobin level from 10.0 g/dL to 10.9 g/dL was classified as mild. In addition, youngsters under five years with a hemoglobin level from 7.0 g/dL to 9.9 g/dL and youngsters with hemoglobin less than 7 g/dL were classified as moderate and severe. Similar anemia categories were used in studies by Muchie et al., Kawo et al., and Habyarimana et al. [25,26,27].

#### 2.2.2. Explanatory Variables

**Figure 1:**
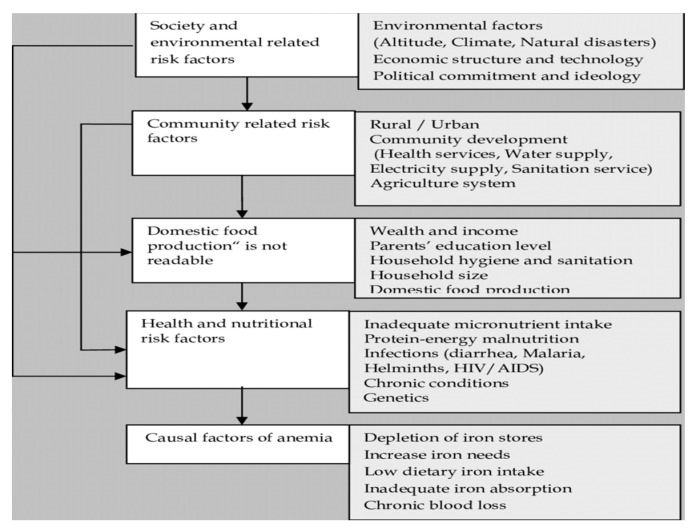
A conceptual framework for the analysis of anemia risk.

Socio-economic, demographic, health and environmental elements of living are known to be the contributing factors into anemia status. The framework (Figure 1) used to select the explanatory variables is similar to that used by Gaston et al., Kawo et al., and Ray et al. [10,26,28].

The community-level variables included in the study is the setting of rural or urban residences. Household-level variables included in the study are household size (0–5, 6–10, 11–15 or >15), sex of household head (male or female), mother’s level of schooling (primary, secondary, or higher), wealth status (poor, middle, or not poor), marital status (unmarried, divorced, married), and birth interval (<24, 24–47 or >47). Wealth status is a combined measure of living standards. It is a calculated index using households’ assets, ownership of living stock, household construction material, and water and sanitation facilities.

Individual-level variables are the gender of the youngster (Male or Female), youngster’s age (0–12, 12–23, 24–35, 36–47, or 48–59 months), youngster birth order (2–3, 4–5, or >5) and youngster nutritional status (mild, moderate, and severe).

The novelty of this study includes employing the malnutrition status variable and socio-economic characteristics. The above were excluded in studies by Ngwira & Kazembe, Kawo et al., and Habyarimana et al. [11,26,27].

#### 2.2.3. Computation of Wealth Index Using Principle Component Analysis (PCA)

Principal component analysis extracts all the factors underlying a set of variables and completely explains the variance in each variable. Component analyses unities (values of 1.0) are inserted in the diagonal of the correlation matrix to ensure that full variance is brought into the factor matrix [29].

In this study, PCA was conducted to create an asset index (which will be used to define household wealth status) using 2016 Senegal data, 2016 Malawi data and 2016 Angola data. The demographic health survey included information regarding the ownership of durable goods, housing characteristics, access to services, along with basic demographics concerning household size and composition.

A standardized index (SI) was developed to classify the household as poor, middle and not poor. The SI formula is given by

$$\frac{\mathrm{NSI}\;\mathrm{of}\;\mathrm{Factor}\;1\;-\;\mathrm{Min}\;\mathrm{NSI}}{\mathrm{Max}\;\mathrm{NSI}\;-\;\mathrm{Min}\;\mathrm{NSI}}$$

where NSI is a non-standardized index, min NSI is the minimum value computed under NSI and Max NSI is the maximum value computed under NSI. The values of SI range from 0 to 1. Therefore, households with a value of less than 0.30 was classified as poor, households with avalue between 0.03 to 0.60 was classified as middle and households with a value greater than 0.60 was classified as not poor. This approach is only use when the sample size is greater than 350 [30].

**Figure 2:**
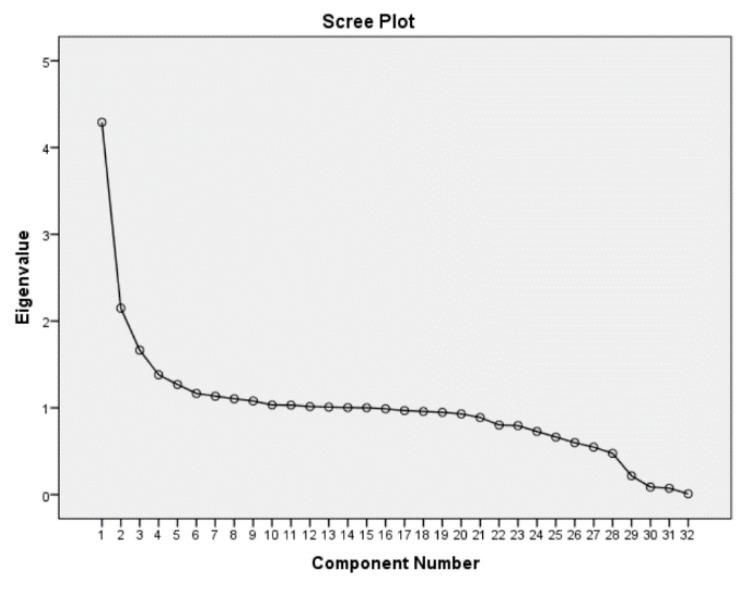
Scree Plot of Senegal DHS data.

**Figure 3:**
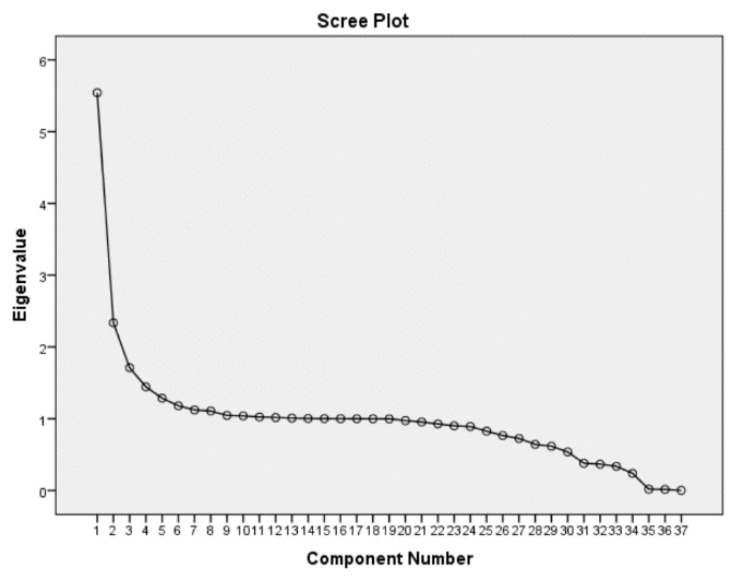
Scree Plot of Malawi DHS data.

**Figure 4:**
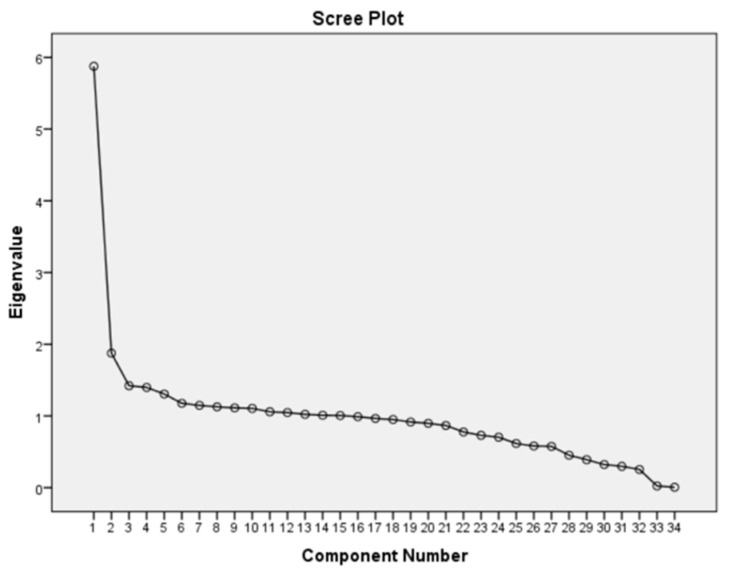
Scree Plot of Angola DHS data.

The scree plots in Figure 2, Figure 3 and Figure 4 display the eigenvalues associated with a component in descending order against the component number. The results from three scree plots indicate that the first three components would be sufficient to explain the original variables. These three components are declared to be sufficient in explaining the original variables because the slope of the curve is leveling off (the elbow) after the third component.

### 2.3. Analyses

#### 2.3.1. Descriptive Analysis

The Chi-square and Goodman gamma measures were employed to test for association between the outcome variable and the explanatory variable. The strength between the outcome variable and the ordinal explanatory variable was tested by employing Goodman gamma measure [31]. Goodman gamma is calculated using the below formula:

(1) $$\frac{\mathrm{Ns}\;-\;\mathrm{Nd}}{\mathrm{Ns}\;+\;\mathrm{Nd}}$$

where Ns is the total number of concordant pairs and Nd is the total number of discordant pairs.

Whereas, the strength between the outcome variable and the nominal explanatory variable was tested by employing chi-square [31]. The formula for chi-square is given by;

Σ [(O-E)/E](2)

where O represents the observed frequency and E is the expected frequency under the null hypothesis.

The Bonferroni correction was applied to make corrections for the number of tests performed.

#### 2.3.2. Multilevel Analysis

Generalized linear mixed models are the models that outspread the class of linear models by including random effects. This type of model outgrows the linear mixed model and generalized linear model of which are known as a special case of generalized linear mixed models. The advantage of using the generalized linear mixed models include the flexibility to analyze non-normal data when random effects are present [32].

The equation form of the generalized linear mixed model is given by

g(u) = g[E(Y|y)] = βX + Zn(3)

where Z is the random effect, β is the fixed effect parameters, and X is the fixed effect covariates.

## 3. Results 

### 3.1. Descriptive Analyses Interpretation

The results of the descriptive analysis were obtained from SPSS (IBM, Armonk, NY, USA) [33]. Table 3 summarizes the findings based on the created pooled sample of youngsters under five years. The *p*-value of all the factors were obtained through Goodman gamma measure and chi-square. The Goodman gamma measure was used for the ordinal covariate, whereas the chi-square test was used for the nominal covariate. The results from Table 1 show that the youngster’s age, type of residence, the gender of the youngster, birth interval, mother’s level of schooling, wealth status, and nutritional status are associated with anemia at 5% level of significance.

There is a high prevalence of youngsters under five years residing in rural settings of Senegal, Malawi, and Angola that are affected by severe and moderate anemia when compared to youngsters under five years residing in urban settings affected by severe and moderate anemia (2.4% and 32.0% respectively, *p*-value = 0.000). It can be observed that a high proportion of youngsters in the age interval of 0–12 months is moderate (49.8%, *p*-value = 0.000). In contrast, a high proportion of youngsters in the age interval of 13–59 months is mildly anemic.

There is a higher proportion of male youngsters that are affected by severe and moderate anemia in Senegal, Malawi, and Angola when compared to their female counter-part (2.5% and 31.4% respectively, *p*-value = 0.000).

There is a high prevalence of under five-year youngsters residing with primary educated mothers that are moderate anemic when compared to under five-year youngsters residing with secondary or higher educated mothers (2.4%, *p*-value = 0.000). In addition, youngsters with shorter birth intervals (<24 months) are more exposed to severe anemia condition when compared to youngsters with longer birth intervals (2.3%, *p*-value = 0.046). Furthermore, youngsters under five years from less fortunate households tend to be affected by severe and moderate anemia when compared to under five years youngsters from a middle and not poor households (3.1% and 33.2% respectively, *p*-value = 0.000). 

There is a high prevalence of youngsters under five years with severe nutritional conditions that are suffering from severe and moderate anemia when compared to children with moderate and mild nutritional and anemia status (3.9% and 36.5% respectively, *p*-value = 0.000).

### 3.2. Multilevel Application

The initial step on model selection was to include all the factors and their interactions into the model. All the factors that were not significant were excluded from the last model. Seven factors were included in the last model:

logit [u] = β_0_ + β_1_ Residence setting_ij_ + _β2_ Youngster’s age_ij_ + β_3_ Gender of the youngster_ij_ + _β4_ Mother schooling_ij_ + β_5_ Birth interval_ij_ + _β6_ Wealth status_ij_ + β_7_ Nutritional status_ij_ + b_i_ + b_ij_ (4)

where β_0_; β_1_; …; β_7_ are the parameter estimates. 

The AIC and BIC information criteria were used to identify whether one random intercept model or two random intercept model is appropriate. The AIC and BIC results showed that one random intercept model is appropriate. Hence, in the final model country is stated as the random factor.

The results of the multilevel analysis were obtained from SAS (SAS Institute Inc.: Cary, NC, USA) [34]. The multilevel analysis table (Table 4) presents the findings correlated with youngster age, residence setting, youngster gender, birth interval, mother schooling, wealth status, and nutritional status. 

The finding shows that youngsters under five years residing in the rural settings of Senegal, Malawi, and Angola are 0.935 more likely to be affected by anemia when compared to youngsters under five years residing in the urban settings of Senegal, Malawi, and Angola (*p*-value = 0.280). This outcome is in agreement with the findings of the study conducted by Ncogo et al. [35].

Regarding youngsters age less than 12 months, it is observed that youngsters in the age interval 13–23, 24–35, 36–47 and 48–59 are more likely to be affected by anemia (OR = 1.419, 2.282, 3.174 and 4.874 respectively). Furthermore, the odds ratio of being affected by anemia among under five-year youngsters is observed to be increasing as the age interval increases.

Female youngsters under five years in Senegal, Malawi, and Angola are 1.197 times at risk of being affected by anemia when compared to their male counterparts (*p*-value ≤ 0.001). 

Youngsters under five years residing in a household with the mother that attained a secondary level of schooling are 1.237 times at risk of being affected by anemia when compared to youngsters under five years residing in the household with the mother that obtained a higher level of schooling. Whereas, youngsters under five years living in the household with the mother that obtained primary level of schooling are 1.111 times at risk of being affected by anemia when compared to youngsters under five years living in the household with the mother that obtained a higher level of schooling.

Youngsters under five years residing in a poor household are 1.310 times more likely to be affected by anemia when compared to youngsters residing in not poor households (*p*-value ≤ 0.001). Whereas, youngsters under five years residing in a middle-classified household are 1.214 times more likely to be affected by anemia when compared to youngsters residing in not poor households (*p*-value = 0.008). 

Nutritional severely and moderate youngsters from Senegal, Malawi, and Angola are at a higher risk of being affected by anemia when compared to youngsters that are nourished (OR = 1.492 and 1.914 respectively).

A type 3 test of fixed effects tests the significance of each fixed effect specified when modeling. The null hypothesis is that the fixed effect variable does not significantly explain the response variable, against the alternative that it does. The results in Table 5 show that youngster age, gender, mother’s level of schooling, wealth status, and nutritional status significantly explain anemia at the 5% level of significance.

## 4. Discussion

In this study, the generalized linear mixed model was utilized to determine the determinants of anemia among youngsters under five years in Senegal, Malawi, and Angola. The Demographic and Health Survey information from Senegal, Malawi, and Angola was merged to create a pooled sample. This statistical technique was also used by Takele et al., Subramanian et al., Neuman et al., and Khulu & Ramroop [21,22,23,24].

The results were obtained from SPSS and SAS software. The descriptive analysis was performed to identify factors associated with anemia in Senegal, Malawi, and Angola. A generalized linear mixed model was performed to identify key determinants of anemia among under five-year youngsters. The results from the generalized linear mixed model showed that youngster age, gender, mother’s level of schooling, wealth status and nutritional status significantly explain anemia among under five-year youngsters in Senegal, Malawi, and Angola.

Multilevel analysis findings show that female youngsters are more likely to be affected by anemia when compared to their male counterparts in Senegal, Malawi, and Angola. Results further reveal that the increase in youngster’s age positively increases the chances of being exposed to anemia. Youngsters of age greater than 13 months are more likely to be affected by anemia. This finding indicates that youngsters transitioning from breastfeeding to normal dietary are more expose to anemia condition.

Mother schooling is found to be significantly explaining anemia. Base on the findings, it can be observed that improving the mother’s level of schooling reduces the risk of being affected by anemia among youngsters under five years. The results revealed that youngsters living with mothers who obtained primary and secondary levels of schooling are at a higher risk of being exposed to anemia. Similar findings were obtained by Tekele et al. [21]. Therefore, Senegal, Malawi, and Angola governments need to improve the schooling strategies and systems to control children’s mortality rate. 

Household wealth status results show that youngsters under five years living in the household that is poor and middle are more expose to anemia. This variable is found to significantly explain the prevalence of anemia. The governments of Senegal, Malawi, and Angola need to focus on how to uplift the population of poor and middle households from an economic perspective through the introduction of financial programs.

Similarly, the findings on the nutritional status indicate that, to control youngsters under five years anemic conditions, the focus must be on severe and moderate youngsters. This is in agreement with the results of the studies conducted by Khan et al. and Zhoa et al. [36,37]. 

## 5. Conclusions

The results of descriptive statistics and multilevel analysis were obtained from SPSS and SAS respectively.

In this study, seven factors were included in the final model. However, only five were found to be significant in explaining anemia at the 5% level of significance. A generalized linear mixed model identified youngster’s age, gender, mother’s level of schooling, wealth status, and nutritional status as determinants of anemia among youngsters under five years in Senegal, Malawi, and Angola. 

Therefore, it can be asserted that under-five year female youngsters between the age of 0 and 59 months with a severe or moderate nutritional status from a poor or middle household and with primary or secondary educated mothers are more exposed to anemia in Senegal, Malawi, and Angola. This finding is in agreement with the findings of the research conducted by Habyarimana et al., Zhoa et al., and Woldie et al. [27,37,38].

To achieve the SGD target, there is a need for Senegal, Malawi, and Angola to initiate programs that will focus on improving the economy of all communities in an attempt to close the gap between poor and not poor households and improve women’s education. 

Furthermore, with the limited resource in the African continent, this suggests the necessity of collaboration between Senegal, Malawi, and Angola to achieve the SGD target. Collaboration between the three countries is pivotal to ensuring that all the identified factors are controlled.

Limitations of the study includes modelling common factors across Senegal, Malawi, and Angola. This reasoning induced the omission of important factors identified by literature to be associated with anemia among youngsters under five years old. Future research must include dietary variables, episodes of diarrhea, etc.

## Figures and Tables

**Table 1:** Pooled sample size breakdown by country.

Country	Severe Anemic	Moderate Anemic	Mild Anemic
Senegal	76	901	1881
Malawi	63	1310	2856
Angola	102	1257	3111

**Table 2:** Summary statistics of pooled sample.

Country	Year	Age of Children (months)	Community Type	Gender	Sample Size
Senegal	2016	0–59	Rural and Urban	Male and Female	2858
Malawi	2015–2016	0–59	Rural and Urban	Male and Female	4229
Angola	2015–2016	0–59	Rural and Urban	Male and female	4470

**Table 3:** Assessing the association between selected covariate and anemia status of under-five youngsters using the gamma and chi-square test, pooled sample.

Factors *	Severe (%)	Moderate (%)	Mild (%)	r (*p*-Value)	χ^2^ (*p*-Value)
Residence setting					
Rural	180 (2.4%)	2396 (32.0%)	4923 (65.6%)	-	**0.000**
Urban	61 (1.5%)	1072 (26.4%)	2925 (72.1%)		
Youngster’s age (months)					
0–12	22 (3.1%)	348 (49.8%)	329 (47.1%)	**0.000**	-
13–23	50 (2.9%)	769 (44.0%)	927 (53.1%)		
24–35	60 (2.4%)	800 (32.3%)	1620 (65.3%)		
36–47	71 (2.2%)	860 (26.1%)	2366 (71.8%)		
48–59	38 (1.1%)	691 (20.7%)	2606 (78.1%)		
Gender of youngster					
Male	147 (2.5%)	1831 (31.4%)	3851 (66.1%)	-	**0.000**
Female	94 (1.6%)	1637 (28.6%)	3997 (69.8%)		
Mother schooling					
Primary	199 (2.4%)	2615 (31.5%)	5498 (66.1%)	**0.000**	-
Secondary	29 (1.3%)	579 (26.8%)	1551 (71.8%)		
Higher	4 (2.6%)	29 (18.6%)	123 (78.8%)		
Birth interval					
<24	20 (2.3%)	224 (26.0%)	616 (71.6%)	**0.046**	-
24–47	85 (2.1%)	1171 (29.2%)	2755 (68.7%)		
>47	61 (1.9%)	1002 (30.7%)	2204 (67.5%)		
Wealth status					
Poor	178 (3.1%)	1921 (33.2%)	3680 (63.7%)	**0.000**	-
Middle	35 (1.4%)	701 (28.3%)	1739 (70.3%)		
Not poor	28 (0.8%)	846 (25.6%)	2429 (73.5%)		
Birth order					
2–3	72 (2.1%)	1024 (29.8%)	2344 (68.1%)	0.221	-
4–5	54 (2.4%)	658 (29.4%)	1525 (68.2%)		
>5	48 (1.6%)	876 (28.9%)	2102 (69.5%)		
Nutritional status					
Severe	76 (3.9%)	717 (36.5%)	1171 (59.6%)	**0.000**	-
Moderate	39 (2.5%)	477 (30.0%)	1073 (67.5%)		
Nourished	126 (1.6%)	2274 (28.4%)	5604 (70.0%)		
Marital status					
Married	16 (2.0%)	219 (28.0%)	548 (70.0%)	-	0.762
Living together	200 (2.1%)	2855 (30.1%)	6432 (67.8%)		
Widowed	23 (2.0%)	352 (30.6%)	776 (67.4%)		

* Wealth status was computed using principal component analysis. Significant factors are highlighted in bold.

**Table 4:** Covariates parameter estimates and Odds ratio using the pooled sample.

Factors *	Est.	Std. Error	OR	*p*-Values
Intercept	−0.685	0.132		0.035
Mild				
Intercept	2.466	0.148		0.003
Moderate				
Residence setting				
Ref: Urban				
Rural	−0.067	0.062	0.935	0.280
Youngster’s age				
Ref: 0–12				
13–23	0.350	0.120	1.419	0.004
24–35	0.825	0.117	2.282	<0.001
36–47	1.155	0.116	3.174	<0.001
48–59	1.584	0.118	4.874	<0.001
Gender of youngster				
Ref: Male				
Female	0.180	0.050	1.197	<0.001
Mother schooling				
Ref: Higher				
Primary	0.105	0.273	1.111	0.702
Secondary	0.213	0.076	1.237	0.005
Birth interval				
Ref: 0–24 months				
24–47	−0.022	0.091	0.978	0.809
>47	−0.083	0.057	0.920	0.144
Wealth status				
Ref: Not poor				
Poor	0.270	0.076	1.310	<0.001
Middle	0.194	0.073	1.214	0.008
Nutritional status				
Ref: Nourished				
Severe	0.400	0.069	1.492	<0.001
Moderate	0.194	0.093	1.214	0.037
McFadden R Squared	0.254			

* Est. = Parameter Estimates, Std. error = Standard error.

**Table 5:** Type 3 Test of fixed effects.

Factors	Num DF *	Den DF *	F Value	*p* Value
Residence	1	7737	1.17	0.280
Youngster’s age	4	7737	80.02	<0.001
Gender of youngster	1	7737	12.88	<0.001
Mother Schooling	2	7737	3.89	0.021
Birthing Interval	2	7737	1.13	0.322
Wealth Status	2	7737	7.15	<0.001
Nutritional Status	2	7737	18.08	<0.001

* Num DF = Numerator degrees of freedom. Den DF = Denominator degrees of freedom.
